# Modeling and Feasibility Assessment of Mineral Carbonation Based on Biological pH Swing for Atmospheric CO_2_ Removal

**DOI:** 10.1021/acssuschemeng.4c10708

**Published:** 2025-05-08

**Authors:** Yukun Zhang, Spencer Long, Manon T. Duret, Liam A. Bullock, Phyllis Lam, Aidong Yang

**Affiliations:** † Department of Engineering Science, University of Oxford, Oxford OX1 3PJ, U.K.; ‡ School of Ocean and Earth Science, University of Southampton, Southampton SO14 3ZH, U.K.; § Department of the Geophysical Sciences, University of Chicago, Chicago, Illinois 60637, United States; ∥ Climate Systems Engineering Initiative, University of Chicago, Chicago, Illinois 60637, United States; ⊥ Geological and Mining Institute of Spain, IGME C/Rios Rosas 23, Madrid 28003, Spain

**Keywords:** atmospheric CO_2_ removal, mineral carbonation, pH swing, sulfur cycle, microbial process, mathematical modeling

## Abstract

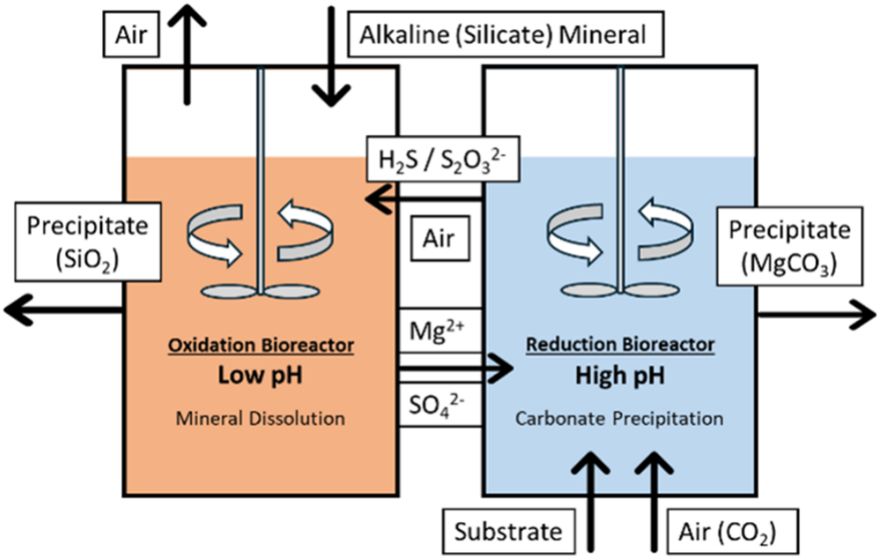

Mitigating climate change requires both the reduction of greenhouse gas emissions and the removal of CO_2_ from the atmosphere. This study investigates a novel biological pH swing strategy for mineral carbonation at ambient conditions as a potential option for atmospheric CO_2_ removal. Through mathematical modeling, we evaluated a mineral carbonation system that utilized *Desulfovibrio vulgaris* and *Acidithiobacillus thiooxidans* to achieve alternating sulfur reduction and oxidation, respectively, with the cyclic process to effect pH swing for promoting the dissolution of a silicate mineral and the subsequent precipitation of a carbonate mineral to store CO_2_. Sulfur cycles employing two reduced compounds, namely, hydrogen sulfide and thiosulfate, were compared. Our simulation results predicted that it is feasible to use the sulfur cycles to achieve the intended pH swing in a range of 1–10 and hence the acceleration of CO_2_ removal from the air. Despite the implementation of the pH swing, gas–liquid mass transfer and mineral dissolution remained rate-limiting compared to biological conversion. Dissolving 35 kg of forsterite in a 1 m^3^ reactor takes between 250 and 300 h, leading to the removal of approximately 22 kg of CO_2_ through MgCO_3_ precipitation, which requires about 180 h. Between the two forms of reduced sulfur, thiosulfate would offer considerable operational advantages over hydrogen sulfide. This theoretical exploration also identified key areas to be investigated to further establish the potential of the sulfur-cycle-based carbonation approach to CO_2_ removal.

## Abstract 2

Schematic of a sulfur-cycle-based biological pH swing system offering a sustainable method for atmospheric CO_2_ removal at ambient conditions.

## Introduction

The elevated concentration of carbon dioxide (CO_2_) in the atmosphere is one of the primary contributing factors to climate change, resulting from human activities such as burning fossil fuels, which currently releases more than 38 gigatonnes of CO_2_ to the atmosphere per year.^1^ To address this pressing issue and limit the increase of global average temperature to 1.5 °C in 2100, a unified international endeavor of deploying innovative carbon dioxide removal (CDR) strategies, in parallel with significant reduction of greenhouse gas emissions, is imperative.^1^

CDR strategies facilitate the removal and long-term sequestration of atmospheric carbon through several mechanisms, including the direct capture of atmospheric CO_2_ via solid or liquid absorption, artificial enhancement of weathering reactions, biological processes that convert CO_2_ into organic compounds (such as biochar), soil carbon sequestration, ocean alkalinity enhancement, and mineral carbonation.^2−6^

Proposed by Seifritz in 1990,^2^ mineral carbonation has been considered as a potentially viable strategy for long-term mitigation of the greenhouse effect. It encompasses two primary methods: (1) in situ mineral carbonation, which involves the direct injection of CO_2_ into basalt and other igneous rocks within the Earth’s crust, with carbonation reactions occurring underground; (2) ex situ mineral carbonation, which occurs above ground through a reactor-based industrial process.^7^ As illustrated by the (direct) carbonation of magnesium silicate shown in eq 1, both types of carbonation involve the chemical reaction of CO_2_ with alkaline minerals, including magnesium, calcium, and iron oxide-based silicates, resulting in the formation of carbonate minerals, which are relatively stable and can store CO_2_ on long-term time scales (>10^6^ years) without the need for continuous monitoring^8^
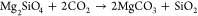
1The process of mineral carbonation is markedly slow under atmospheric temperature and pressure conditions, and the corresponding time frame for reactions would be measured in millennia, which does not allow for atmospheric carbon to be removed on time scales relevant to mitigating climate change.^9^ This problem is primarily caused by two rate-limiting processes. The first one is mineral dissolution, where the inherent low rate is related to the stability of the chemical bonds within the mineral and the available reactive surface area. Usually, larger particles possess a lower specific surface area, which significantly reduces the mineral dissolution rate, especially under the mildly acidic conditions typically found in nature. Second, the relatively low concentration of CO_2_ in the atmosphere leads to severely restricted CO_2_ mass transport and further slows the carbonation reaction. These rate limitations are commonly observed across the carbonation of various silicate minerals.^10−13^ To expedite the carbonation process and amplify mineral reactivity, mineral carbonation procedures often incorporate pretreatments (such as mechanical grinding for reducing the size of mineral particles), alongside the use of elevated temperatures and pressures.^10−12^ This was illustrated by the carbonation of the silicate mineral wollastonite (CaSiO_3_), where particles at a size smaller than 38 μm were treated at 175 °C and a CO_2_ pressure of 10–40 bar, reducing the carbonation time to below 30 min.^14^ Operating the carbonation process at such elevated conditions inevitably leads to engineering and cost challenges, consequently reducing net carbon removal and hindering the feasibility of large-scale application.^15^

In this study, we assess an alternative approach to mineral carbonation, which is to operate at ambient temperature and pressure conditions suitable for CDR from air. This approach implements indirect carbonation. In contrast to direct carbonation introduced in eq 1 where carbonation occurs in a single step, an indirect process involves the dissolution of a silicate mineral first, with the resulting solution undergoing a subsequent carbonation process that leads to the precipitation of a solid carbonate that stores CO_2_. The acceleration of the conversion process in the proposed approach is through sulfur-cycle-based biological pH swing, which has the potential to achieve rapid mineral carbonation without operating under high-temperature and high-pressure conditions. The use of pH swing to effect indirect carbonation through mineral dissolution and precipitation has previously been investigated using acidic and alkaline solutions prepared through potentially costly inorganic processes (e.g., ref (16)). The new process explored in the current work involves the creation of a low-pH environment by microbial sulfur oxidation and the creation of a high-pH environment by microbial sulfur reduction. In this analysis, we selected forsterite as the model mineral. Compared with other Mg-rich silicate minerals, forsterite readily dissolves under acidic conditions, releasing Mg^2+^ ions that react with CO_2_ to form stable carbonate minerals. The desirable dissolution kinetics of forsterite, combined with its global abundance and ease of industrial extraction, makes it an ideal candidate for our process.^17,18^ Primarily through mathematical modeling, this explorative study aims to establish the technical feasibility of this novel approach, in terms of the attainable degree of pH swing and rate of mineral dissolution and precipitation. The findings of this study carry implications for the viability of bioreactor-based CDR approaches, particularly relevant in industrial contexts that utilize alkaline feedstocks, such as mining and steelmaking operations.

## Methods

### Overview of Sulfur-Cycle-Based Biological pH Swing

Sulfur-cycle-based biological pH swing refers to utilizing metabolic processes of microorganisms to implement the oxidation and reduction of sulfur, releasing and consuming, respectively, protons (H^+^) and consequently modulating the pH within the process. The idea of using a biological cycle to effect pH swing is based on the proposal by Salek et al.^19^ and the GGREW project.^20^

In this study, we evaluate two reduced forms of sulfur, which could be involved in biological conversions:^21^ hydrogen sulfide (H_2_S) and thiosulfate (S_2_O_3_^2–^), both can be oxidized to or reduced from sulfate (SO_4_^2–^). Biologically reducing sulfate predominantly to thiosulfate as opposed to hydrogen sulfide has not been widely investigated, although this could be made possible through, for example, disrupting genes responsible for thiosulfate reduction in a sulfate reducer.^22^ The chemical equations for these two cycles are presented by eqs 2, 3 and 4, 5, respectively
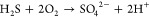
2
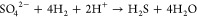
3
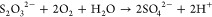
4
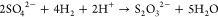
5

**Figure 1:**
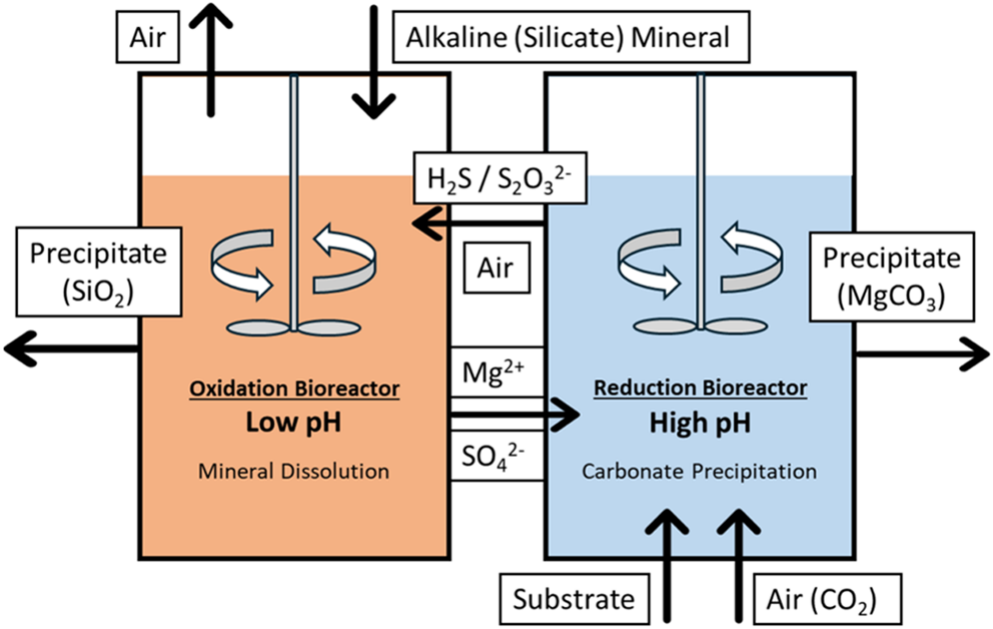
Scheme of the sulfur-cycle-based biological pH swing system.

As shown in Figure 1, the overall system comprises two primary components: the oxidation bioreactor and the reduction bioreactor. Within the oxidation bioreactor, which is aerated for oxygen supply, microbes metabolically oxidize hydrogen sulfide (H_2_S) or thiosulfate ions (S_2_O_3_^2–^) to sulfate ions (SO_4_^2–^). This reaction supplies energy to sustain microbial activities while reducing the pH and hence enhancing the dissolution rate of magnesium-containing alkaline minerals in the bioreactor. For the magnesium-containing alkaline minerals, forsterite (Mg_2_SiO_4_), which is the magnesium-rich member of olivine, was selected as a model silicate because of its relatively high abundance and high reactivity under acidic environment.^17,18,23^ The process of forsterite dissolution can be described by the following chemical equation^24^
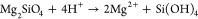
6On the other side of the system, the reduction bioreactor receives Mg^2+^ ions and SO_4_^2–^ ions produced in the oxidation bioreactor. Within the reduction bioreactor, microbes are employed to reduce SO_4_^2–^ ions to H_2_S or S_2_O_3_^2–^ ions. By controlling the amount of electron donors supplied, this process elevates the pH to 10, facilitating the absorption of CO_2_ (at atmospheric concentration) introduced by the air feed into the bioreactor and the reaction between Mg^2+^ ions and dissolved CO_2_, thereby accelerating the precipitation of magnesium carbonate (e.g., magnesite; MgCO_3_), as shown in eqs 7–10
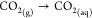
7
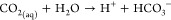
8
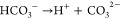
9
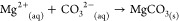
10H_2_S or S_2_O_3_^2–^ ions produced in the reduction bioreactor are subsequently transported to the oxidation bioreactor, initiating the next cycle of the process.

Both bioreactors are agitated and assumed to be well-mixed. Their models are presented in the following sections. In addition, our study incorporates the chemical equilibria of the CO_2_–H_2_O system and the charge balance equation for the pH calculation. Details of these equations and the values of all model parameters are provided in the Supporting Information (SI, Sections S1 and S13). The homogeneous CO_2_–H_2_O reactions are modeled by chemical equilibria because of their fast kinetics, leading to a set of algebraic equations that additionally include charge balance. In contrast, the dynamics of the slow processes such as microbial growth and biochemical conversions, mineral dissolution, and precipitation and gas–liquid mass transfer are captured through ordinary differential equations (ODEs).

Thus, the overall model for each reactor is formulated as a coupled differential–algebraic system, which is solved using the numerical solver ode15s in MATLAB.

### Modeling of the Oxidation Bioreactor

To accelerate the dissolution of forsterite and therefore the release of Mg^2+^ ions, a low-pH environment was preferred in the oxidation bioreactor. *Acidithiobacillus thiooxidans* (*A. thiooxidans*), a mesophilic and chemolithoautotrophic bacterium known for its ability to facilitate sulfur oxidation, has the potential to create an acidic environment and is used here as an example for our model.^25^

#### Microbial Growth Kinetics

First, considering the microbial kinetics, we adopted a modified Monod–Gompertz kinetic model to simulate the proliferation of *A. thiooxidans*(26)
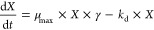
11Equation 11 shows the method to calculate the rate of the net biomass change  (mg h^–1^ L^–1^), where μ_max_ (h^–1^) is the maximum specific growth rate, *X* (mg-dry weight L^–1^) is the microbial density in the liquid phase, *k*_d_ (h^–1^) is the decay rate of *A. thiooxidans*, and γ (—) is the specific growth rate modifier, which can be calculated in eq 12
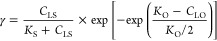
12In this equation, *K*_S_ (mg L^–1^) refers to the half saturation constant of the reduced sulfur compound as the substrate (H_2_S or S_2_O_3_^2–^), *K*_O_ (mg L^–1^) is the kinetic constant of oxygen for microbial growth, and *C*_LS_ and *C*_LO_ (mg L^–1^) are the concentrations of the substrate and DO in liquid, respectively, which could be further calculated with the equations of mass balance introduced below.

#### Gas- and Liquid-Phase Mass Balance and Mineral Dissolution


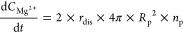
13

Considering the Mg^2+^ ions generated from forsterite dissolution, as shown in eq 13, the rate of change in the overall Mg^2+^ ion concentration depends on the dissolution rate *r*_dis_ (mol m^–2^ h^–1^), forsterite particle radius *R*_p_ (m), and the number of forsterite particles *n*_p_ (—). Equation 14 is applied to estimate the shrinkage of forsterite particles during the dissolution process
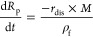
14where *M* (mg mol^–1^) refers to the molar mass of forsterite, and ρ_f_ (mg m^–3^) refers to the average density of forsterite particles applied in the bioreactor.

To calculate the specific dissolution rate of forsterite particles *r*_dis_ (mol m^–2^ h^–1^) at 298.15 K, eq 15 is used^27^
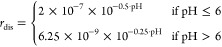
15For gas- and liquid-phase mass balance in the oxidation bioreactor, we developed a liquid-phase mass balance model to describe the dissolution processes of H_2_S and oxygen at the gas–liquid interface, with the dissolution rates determined by volumetric mass-transfer coefficients and Henry’s law constants. These are integrated with the sulfur oxidation kinetics to account for the changes in the concentrations of substrate and dissolved oxygen. Additionally, a gas-phase mass balance model was established, taking into account parameters such as gas flow rate, effective gas volume, and fractional gas hold-up. For detailed equations, please refer to Sections S9 and S10 in the SI.

### Modeling of the Reduction Bioreactor

The reduction bioreactor serves the purpose of absorbing CO_2_ from air and subsequently precipitating MgCO_3_. It receives the influent liquid containing Mg^2+^ and SO_4_^2–^ ions from the oxidation bioreactor, as well as the feed air as the source of CO_2_. To achieve the CDR, an elevated pH environment is necessary to facilitate the formation of MgCO_3_. For this purpose, one sulfate-reducing bacterium in the family of Thermodesulfobacteria, *Desulfovibrio vulgaris* (*D. vulgaris*), is adopted under an anaerobic environment to consume protons and increase pH in the system.^28^

The following modeling methods were applied to the reduction reactor in both the hydrogen sulfide cycle and the thiosulfate cycle.

#### Microbial Kinetics

As shown in previous eq 3, the growth of *D. vulgaris* consumes hydrogen (H_2_) as the reductant to reduce SO_4_^2–^ ions, a process providing the energy source to microbial growth.^29^ To simulate this process in our model, we modified the method introduced by Smith^30^ and applied the following equations to calculate the net biomass change  (g h^–1^ L^–1^) and rate of sulfate consumption *r*_S_ (mol L^–1^ h^–1^)
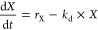
16
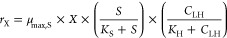
17
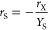
18In these equations, *r*_X_ (g L^–1^ h^–1^) refers to the biomass growth rate, *X* (g L^–1^), μ_max,S_ (h^–1^), and *k*_d_ (h^–1^) correspondingly refer to the concentration, maximum growth rate, and decay rate of *D. vulgaris*. *S* and *C*_LH_ (mol L^–1^) represent the concentrations of sulfate and hydrogen in liquid, respectively. *K*_S_ and *K*_H_ (mol L^–1^), respectively, refer to the Monod constants for sulfate and hydrogen. *Y*_S_ (g L^–1^ mol^–1^) refers to the biomass yield of *D. vulgaris* during sulfate reduction.^31^

#### Gas- and Liquid-Phase Mass Balance and MgCO_3_ Precipitation

For precipitation of MgCO_3_, we employ eq 19 to calculate the saturation index Ω (—) and subsequently use eq 20 to simulate the precipitation rate *r*_prec_ (mol m^–2^ h^–1^) based on the calculated Ω
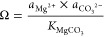
19
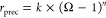
20where *K*_MgCO_3__ (—) refers to the equilibrium constant of MgCO_3_, *a* (—) refers to the activities of participating ions, and *k* (mol m^–2^ h^–1^) refers to the specific rate constant. The precipitation was assumed to be seeded and was dominated by the growth of the solid seeds with an initial total surface area of 1 m^2^ L^–1^. Then, with the total surface area of seeds *A* (m^2^ L^–1^), eq 21 is used to calculate the rate of CO_2_ capture *r*_cap_ (mol L^–1^ h^–1^)
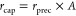
21Furthermore, eq 22 is used to calculate the rate of change in the total surface area *r*_A_ (m^2^ L^–1^ h^–1^)
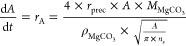
22where *M*_MgCO_3__ (g mol^–1^) and ρ_MgCO_3__ (g m^–3^) refer to the molar mass and average density of MgCO_3_, respectively, and *n*_s_ refers to the number of crystal seeds provided to the bioreactor.

For gas- and liquid-phase mass balance in the reduction bioreactor, we assumed that hydrogen is supplied in a liquid phase and developed separate mass balance equations for the reduced H_2_S or S_2_O_3_^2–^ ions as well as for CO_2_ capture. For detailed equations, refer to Sections S11 and S12 in the SI.

### Validation of the Oxidation Reactor Model

Purposefully executed experiments for validating the bioreactor models described above with exactly the same parameters were not available in this study. However, it was possible to access data from a past experimental study on the oxidation of thiosulfate by *A. thiooxidans* for dissolving silicate mineral-rich mine tailings, which share similarities with the oxidation bioreactor modeled in this work. To gain a certain degree of validation, we adjusted the model accordingly to fit it to the experimental conditions and then compared the simulation results with experimental data. This process includes verifying pH variation and mineral dissolution within the oxidation bioreactor of the thiosulfate cycle.

#### Experimental Setup

The experiment was performed under ambient conditions with no active gas input, and the oxidation bioreactor had a starting volume of 200 mL. Cultures were incubated statically in an open system, and gas–liquid mass transfer occurred via natural diffusion between the media and the atmosphere. The minerals used in the dissolution experiment were 10 g of a sample from mine tailings with an average particle radius of approximately 50 μm. The major compositions of the mine tailings include plagioclase, clinopyroxene, orthopyroxene, serpentine, talc, amphibole, chlorite, mica, quartz, calcite, and dolomite. More details of the culture medium and mine tailings are provided in the SI (Sections S2 and S3).

#### Adaptation of the Model

In contrast to forsterite as a single mineral, the mine tailings employed in the experiment comprise various mineral phases (Table S5), thus requiring separate simulations. We utilized the following modified Arrhenius equation to calculate rates of dissolution of major minerals within the tailings that lead to the release of primarily magnesium and calcium ions
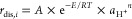
23In this equation, *r*_dis,*i*_ (mol m^–2^ h^–1^) refers to the rate of dissolution for mineral *i* in the mine tailings, *A* (mol m^–2^ h^–1^) refers to the Arrhenius factor of this specific mineral, *E* (J mol^–1^) refers to the molar activation energy, *R* (J K^–1^ mol^–1^) refers to the universal gas constant, *T* (K) refers to the temperature, *a*_H^+^_ (—) refers to the activity of hydrogen ions, and *n* (—) refers to the reaction order.

To simplify the calculation, we assumed that the initial radius of the mine tailings particles was consistent and each particle contained only one type of mineral; then, we modeled the rate of change in surface area (via radius, under the assumption of spherical particles) for various mineral particles during dissolution and combined it with the dissolution rate, as depicted in eqs 24 and 25
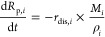
24
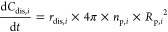
25For mineral *i*, *R*_p,*i*_ (m) refers to its particle radius, *M*_*i*_ (g mol^–1^) refers to the molar mass, ρ_*i*_ (g m^–3^) refers to the density, *C*_dis,*i*_ (mol L^–1^ h^–1^) refers to the overall rate of dissolution in the oxidation bioreactor for this mineral, and *n*_p,*i*_ (L^–1^) refers to the number of mineral particles.

## Results and Discussion

### Comparison of the Results of the Adapted Oxidation Bioreactor Model with Experimental Data

**Figure 2:**
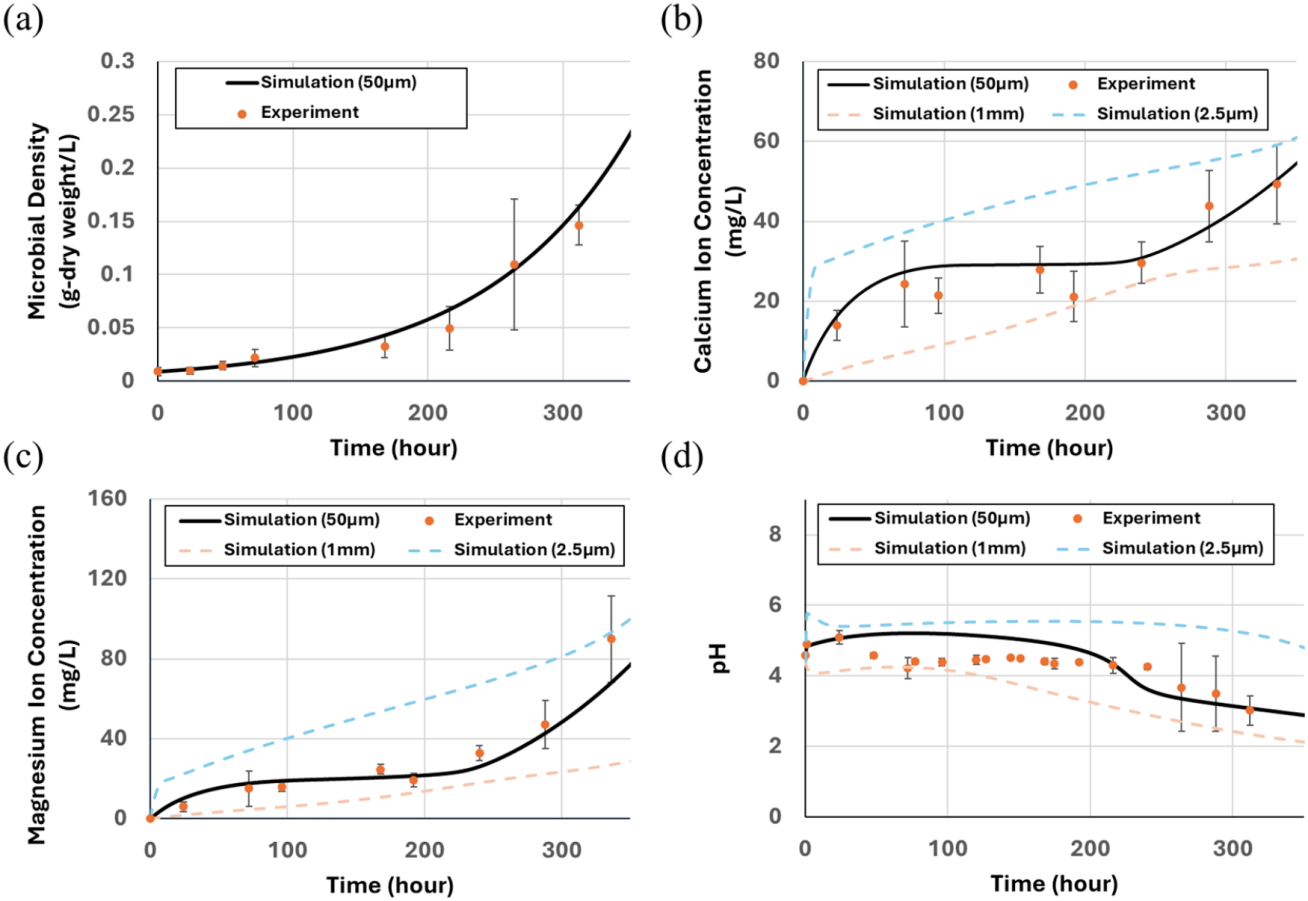
Comparisons between simulation results and experimental data: (a) microbial density (g L^–1^); (b) calcium ion concentration (mg L^–1^); (c) magnesium ion concentration (mg L^–1^); and (d) pH value. Simulation results in panels (b)–(d) are significantly affected by the mineral particle radius, where the predictions at 50 μm (the known average) and two extremes (1 mm and 2.5 μm) are shown.

Figure 2 depicts the comparisons between our simulation results and the experimental data in several aspects: the growth of *A. thiooxidans*, the concentrations of magnesium ions and calcium ions released from dissolved mine tailings, and the pH level.

Prior to simulation, the maximum specific growth rate of *A. thiooxidans* (μ_max_ in eq 11), unavailable from the literature for growth in an environment involving mine tailing dissolution, was calibrated (to 0.0117 h^–1^) against the experimentally measured bacterial growth data (Figure 2a). Mineral dissolution (and hence the pH level) significantly depends on the mineral particle size distribution (PSD). Compared to larger particles, smaller particles possess higher specific surface areas and thus exhibit faster dissolution. This leads to a more rapid release of metal ions and greater counterbalancing against the pH-lowering effect of sulfur oxidation. While the precise PSD of the tailing samples used in the experiments was not available, the effects were reflected through simulations at three particle sizes: the known average particle radius (50 μm) and two hypothetical extreme values (2.5 μm and 1 mm), as presented in Figure 2b–d in comparison with experimental measurements. Overall, our model was shown to be able to reasonably capture the main process behaviors, which lends support for the subsequent use of the modeling framework for simulation studies.

### Results of Simulating Sulfur Cycles for Forsterite Carbonation

The simulations for both the oxidation bioreactor and the reduction bioreactor were completed by using the ode15s solver in MATLAB. Both bioreactors were assumed to have a reaction volume of 1 m^3^ and an inner diameter of 1.13 m; the temperature and pressure were set to 25 °C and 1 atm, respectively. Both reactors were agitated at 100 rpm; the diameter and width for impeller were 0.376 and 0.075 m, respectively. The initial conditions adopted for each simulation run are provided in the SI (Section S4).

#### Results of the Hydrogen Sulfide Cycle

In the oxidation bioreactor of the H_2_S cycle (as well as the S_2_O_3_ cycle), we initially introduced 200 g L^–1^ of forsterite with a particle radius of 50 μm. Additionally, we supplied a total of 0.5 mol of H_2_S gas that was sourced directly from the reduction bioreactor. The total flow rate of the feed gas mixture was 360 m^3^ h^–1^; the supply duration of this gas flow and the change in the concentration of H_2_S were determined by the reduction reactor (see Figure 4).

**Figure 3:**
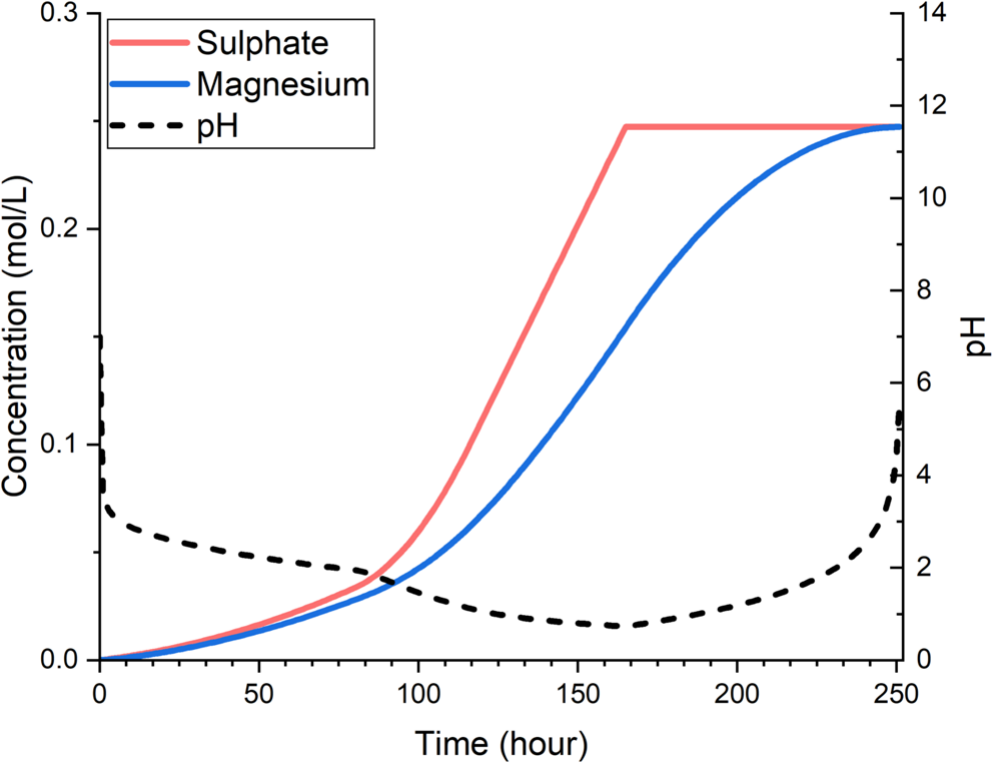
Changes in pH and concentrations of sulfate ions and magnesium ions (mol L^–1^) in the oxidation reactor operating in the H_2_S cycle.

As shown in Figure 3, at the time of approximately 165 h, the oxidation bioreactor completed the oxidation of H_2_S, coinciding with the concentration of sulfate in the solution reaching its maximum. At the same time, the pH level of the solution dropped to its lowest level, prompting forsterite to dissolve and release magnesium ions at an accelerated rate. By approximately 250 h, the dissolution of forsterite neutralized the acidity in solution and enabled the pH value to return to neutral, by which this oxidation stage of the cycle was completed.

During the operation of the oxidation stage, only partial absorption of the H_2_S gas was predicted due to the limitation in mass transfer under the simulated conditions. Therefore, only approximately 0.25 mol of sulfate ions were generated, which represents half of the H_2_S supplied. This observation suggests the need for additional measures such as the recirculation of the H_2_S gas exiting the oxidation bioreactor to reduce the loss of sulfur in the cycle, increase the dissolution of forsterite, and avoid harmful environmental impacts such as corrosiveness and toxicity. However, the process of recirculation would extend the operational duration of the oxidation stage, which was not simulated in this study.

**Figure 4:**
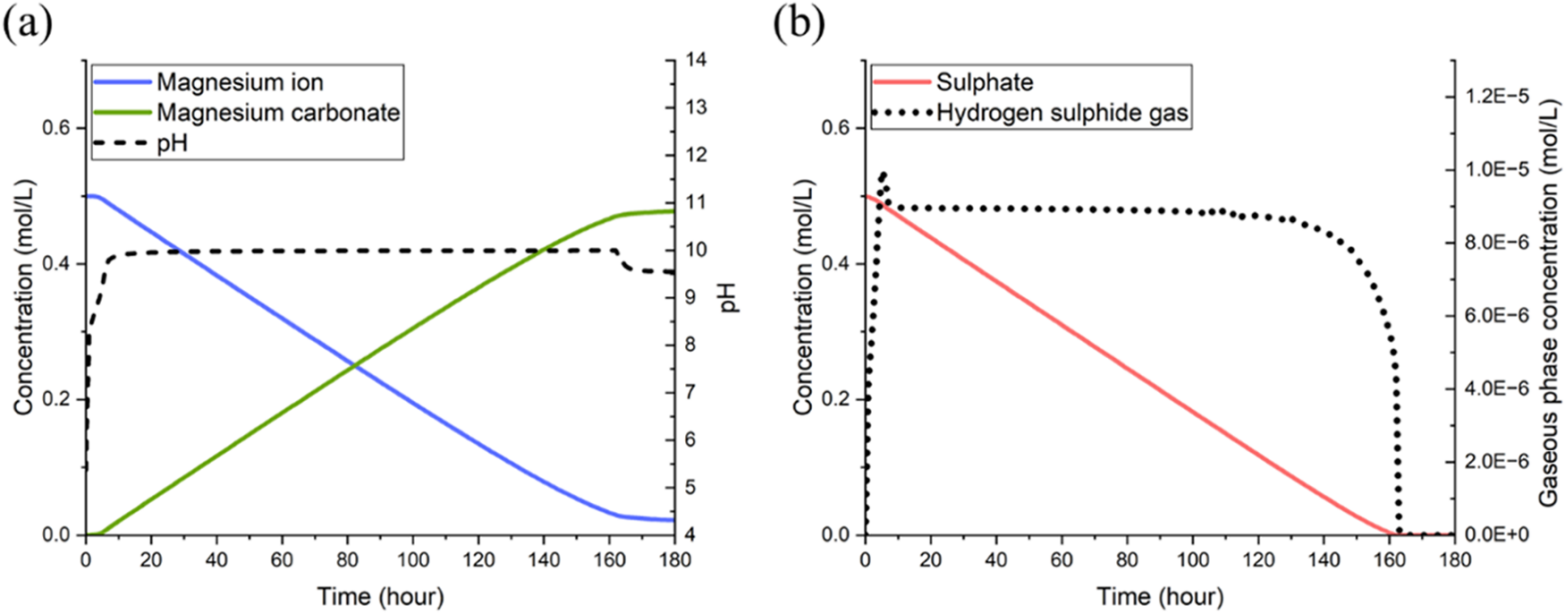
Simulation results for the reduction reactor operating in the H_2_S cycle: (a) changes in pH, magnesium ion concentration in solution, and precipitation of magnesium carbonate (mol L^–1^); (b) aqueous concentrations of sulfate ions and H_2_S concentration in the output gas flow (mol L^–1^).

For the reduction bioreactor, Figure 4 shows that the reduction of sulfate was completed within 170 h and coincided with a significant increase in the pH of the solution. As already stated earlier, in our simulations, we limited the supply of hydrogen gas to restrict the metabolic rate of microorganisms, thereby keeping the solution pH below 10. This approach was aimed at preventing the undesirable formation of Mg(OH)_2_ and ensuring high utilization of magnesium ions in the carbon capture process. Additionally, it is noteworthy that restricting the solution pH does not significantly reduce the concentration of CO_3_^2–^ ions, thereby maintaining the efficiency of MgCO_3_ precipitation. Compared with that of pure water, the higher salinity of the solution used in our simulations alters the solution chemistry, allowing it to maintain a relatively high ratio of carbonate ions even at lower pH levels.

Then, depicting the operation of both reactors, Table 1 shows that running one H_2_S cycle requires approximately 251 h, with the reduction bioreactor capturing about 21.75 g L^–1^ of atmospheric CO_2_. As mentioned earlier, additional absorption of H_2_S into the oxidation reactor would still be required, beyond what has been simulated in this work, to enable a complete cycle with a minimum loss of sulfur.

**Table 1:** Timetable for Operation of Carbon Mineralization Based on H_2_S Cycle Biological pH Swing

reactor	process	start time (h)	end time (h)
oxidation bioreactor	oxidation of H_2_S	5	165
	dissolution of forsterite	5	251
reduction bioreactor	H_2_S gas generation	0	164
	MgCO_3_ precipitation	6	169

#### Results of the Thiosulfate Cycle

**Figure 5:**
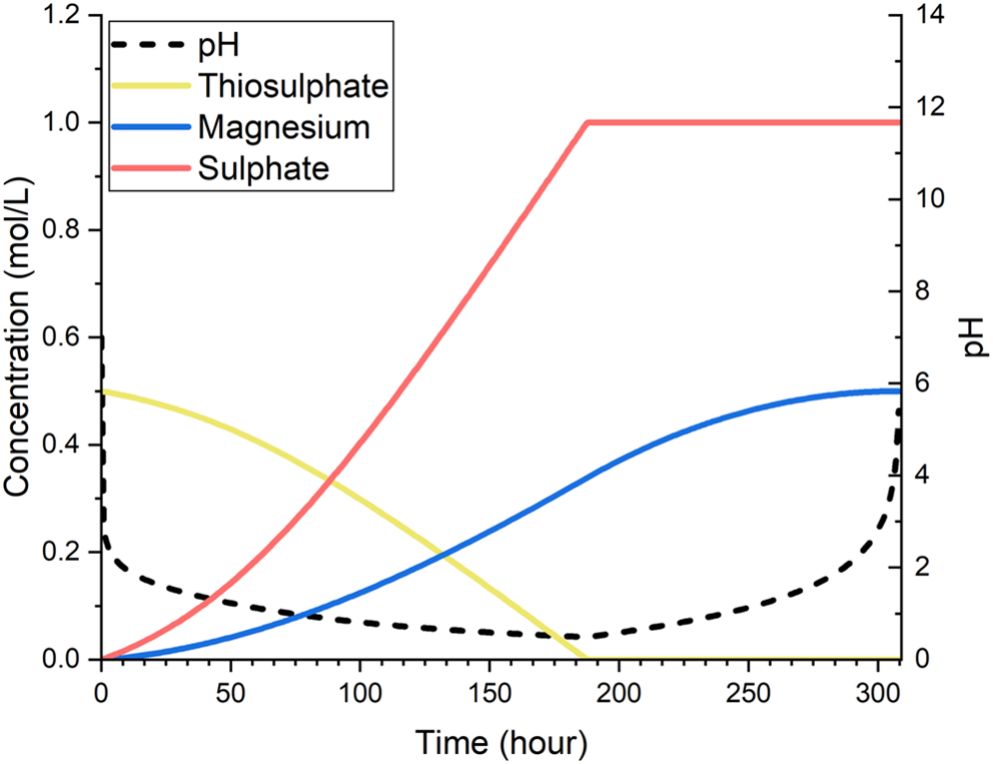
Changes in pH and concentrations of sulfate ions, magnesium ions, and thiosulfate ions in solution (mol L^–1^) in the oxidation reactor operating in the S_2_O_3_ cycle.

Unlike the H_2_S cycle, the oxidation bioreactor of the S_2_O_3_ cycle operates not with a gaseous sulfur input but with the liquid solution containing thiosulfate, which is to be produced by the reduction reactor. As shown in Figure 5, the oxidation of thiosulfate to sulfate lowered the pH to facilitate the dissolution of forsterite. 0.5 mol L^–1^ of thiosulfate was completely oxidized to obtain 1 mol L^–1^ of sulfate within 180 h, which exhibited a significantly higher sulfur utilization rate than its counterpart in the H_2_S cycle simulated. This higher conversion further translated to greater dissolution of forsterite, leading to a 0.5 mol L^–1^ release of magnesium ions.

**Figure 6:**
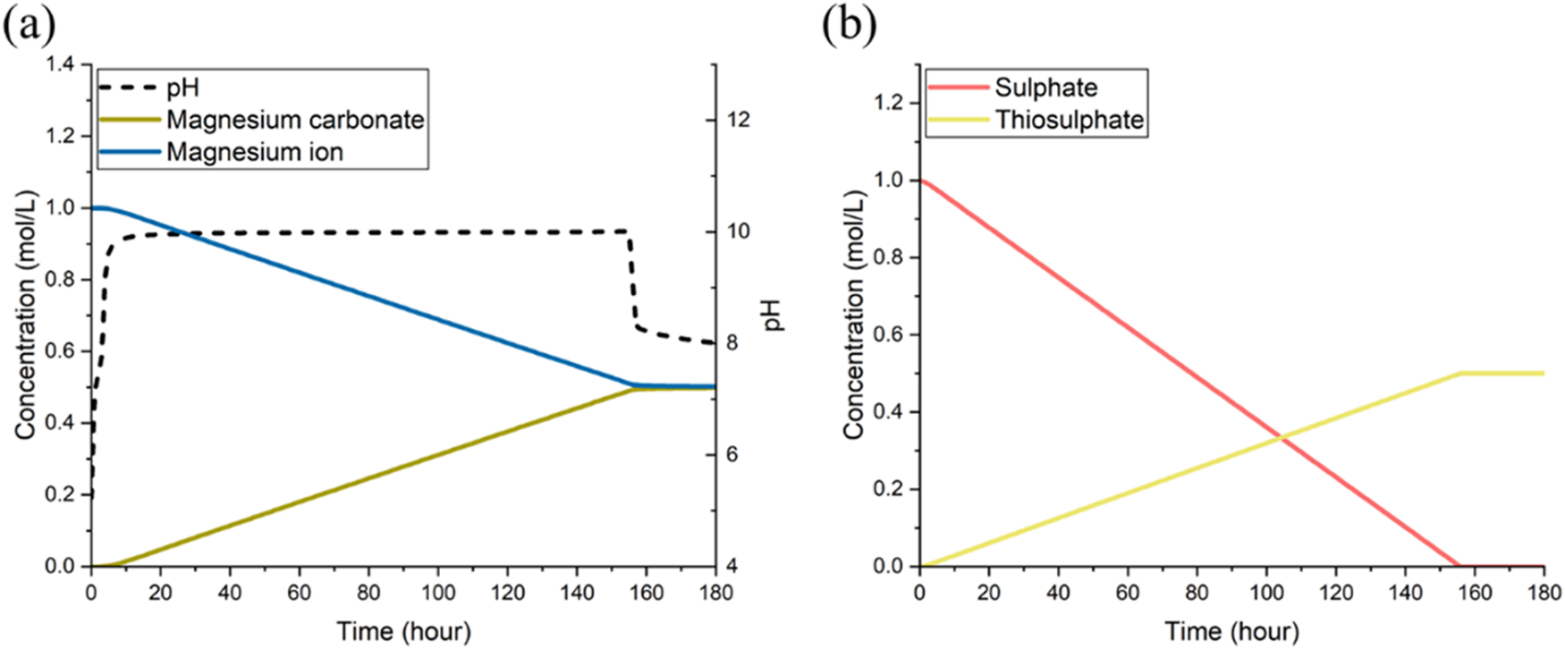
Simulation results for the reduction reactor operating in the S_2_O_3_ cycle: (a) trends of pH, magnesium ion concentration in solution, and precipitation of magnesium carbonate (mol L^–1^); (b) trends of sulfate ion consumption and thiosulfate ion generation (mol L^–1^).

On the reduction side, the conversion of sulfate to S_2_O_3_^2–^ is also simpler than the H_2_S cycle since it does not generate the reduced product in the gas phase. This also means that instead of feeding the reduced sulfur compound from the reduction reactor to the oxidation reactor while it is being generated, moving the liquid solution containing thiosulfate to the oxidation reactor takes place after the reduction batch (including the CO_2_ capture process) is completed. Otherwise, the results of the reduction reactor simulation are similar to those in the H_2_S cycle, as shown in Figure 6.

Depicting both reactors of the thiosulfate cycle, Table 2 shows an indicative operational schedule, which completes one cycle in about 308 h. The CDR rate in reduction bioreactor is very similar to that of the H_2_S cycle, approximately 21.99 g L^–1^ or ∼0.5 mol L^–1^, which is as expected from stoichiometry. However, as mentioned above, this cycle does not suffer from the issue of insufficient conversion in the oxidation stage and thus represents a better implementation of the sulfur-cycle concept.

**Table 2:** Timetable for Operation of Carbon Mineralization Based on S_2_O_3_ Cycle Biological pH Swing

reactor	process	start time (h)	end time (h)
oxidation bioreactor	oxidation of S_2_O_3_	0	188
	dissolution of forsterite	0	308
reduction bioreactor	S_2_O_3_ generation	0	156
	MgCO_3_ precipitation	3	157

### Further Discussion

#### Influence of Various Processes on Cycle Time

Our simulation results have shown that, to oxidize 0.5 mol L^–1^ of H_2_S or S_2_O_3_^2–^ under given conditions (1 m^3^ of reaction volume, 25 °C and 1 atm), completing one cycle takes approximately 300 h. In the oxidation bioreactor, forsterite dissolution appears to be much more time-consuming than that of biological conversion: despite the low pH in the bioreactor, which already significantly increases the dissolution rate of forsterite compared to that at a more neutral pH, it still takes about 250 h to complete the dissolution.

To further examine how cycle times are affected by key factors, including microbial growth rate, concentration of substrates, mineral particle radius, and *k*_L_*a* (by varying the impeller speed), we additionally performed a sensitivity analysis (with the detailed results provided in the SI, Section S5). It shows that the mineral particle radius and impeller speed do not alter the amount of CO_2_ removal per cycle but significantly impact the cycle time of both the H_2_S and S_2_O_3_ cycles, while the minimum influence is caused by the change in microbial growth rate. These results are fully in line with the observations discussed above. The increase in the concentration of substrates leads to prolonged processes, as a greater amount of conversion (reduction or oxidation) is needed, although more CO_2_ removal is achieved by these longer processes; the combined effect of these two trends means that CO_2_ removal per unit time is not significantly affected. To further accelerate the process of releasing Mg ions, grinding forsterite particles to smaller sizes could help by increasing surface area, which, however, will incur a greater energy cost.^8^ Besides mineral dissolution, the major rate-limiting processes lie in the gas–liquid mass transfer of CO_2_ and the precipitation of MgCO_3_ both of which are affected by the low concentration of CO_2_ in air, which represents a fundamental challenge for CDR. To overcome the mass-transfer bottleneck in the CO_2_ capture process, possible measures include further increasing the impeller speed or using enzymes, such as carbonic anhydrase (particularly when it can be sourced economically), to accelerate the CO_2_ hydration reaction, thereby shortening the cycle time.^32,33^

The key function of the biological processes considered in this study was to implement pH swing, which can also be achieved by electrochemical processes such as electrodialysis (ED) and bipolar membrane electrodialysis (BPMED).^34−36^ Compared with the biological approach, electrochemical methods can be more easily integrated with renewable energy sources and are capable of achieving broader pH swings in a shorter period of time. This is particularly advantageous in overcoming one of the primary rate-limiting factors identified in our study: the slow dissolution rate of Mg-rich silicate minerals, where the range of pH swing is constrained by the limited pH tolerance of the microbes employed in biological systems. However, the construction of such electrochemical systems would incur material costs for the membranes and electrodes. Besides, long-term operation of electrochemical systems, especially under conditions requiring a high-pH swing range, may face challenges such as membrane and electrode degradation and scaling issues.^35^ Finally, these two different approaches are likely associated with rather different energy requirements, which need to be better understood in future studies.

Furthermore, it is important to note that oxygen intrusion—caused by air introduced into the reduction bioreactor as well as the residual dissolved oxygen from the oxidation bioreactor—may negatively affect the activity of *D. vulgaris* within the reduction bioreactor. However, the impact of a reduced microbial growth rate on the reactor performance is not expected to be severe.^37−41^ This is because in our study, we needed to deliberately limit the supply of electron donors (hence limiting microbial growth) to maintain an optimal pH, thereby preventing unnecessary magnesium hydroxide precipitation and ensuring high magnesium ion utilization. As a result, even if the activity of *D. vulgaris* is partially inhibited, its impact on the overall CO_2_ capture efficiency remains relatively limited (which is also evident from the sensitivity analysis result presented above). Nevertheless, if further minimization of oxygen interference is required, this may be achieved by separating the reduction bioreactor into a microbial reduction chamber and a precipitation chamber, with only the precipitation chamber exposed to air. This configuration helps to maintain a low dissolved oxygen concentration in the microbial reduction chamber. Additionally, adjustments in the oxidation bioreactor—such as optimizing air flow rates and *A. thiooxidans* concentration—can further reduce the residual dissolved oxygen transferred to the reduction bioreactor.

#### Selection of Energy Source for Sulfur Reduction

Unlike the oxidation stage where the conversion of the reduced sulfur compound (H_2_S or S_2_O_3_^2–^) itself provides the energy needed for the microbial process, the reduction bioreactor requires the supply of an electron donor to sustain microbial growth and sulfur reduction. As depicted in eq 26, in the common practices of *D. vulgaris* cultivation, lactate is often provided and then converted into acetate and hydrogen
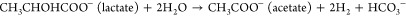
26Subsequently, the generated hydrogen is utilized by *D. vulgaris* for SO_4_^2–^ reduction to provide energy for growth.^42^ While lactate can support the growth of *D. vulgaris*, its conversion involves the release of bicarbonate, which would compete with atmospheric CO_2_ in the precipitation of MgCO_3_, thus hindering the removal of the latter as the intended source feed for mineral carbonation. To circumvent this issue, we opted to supply hydrogen gas directly into the reduction bioreactor. Existing research demonstrates that while lactate is typically the preferred electron donor for the growth of *D. vulgaris*, these bacteria can also directly utilize hydrogen gas to grow and maintain their metabolic functions: in environments devoid of lactate, *D. vulgaris* can utilize other carbon sources, such as acetate, and then switch to using hydrogen gas to reduce sulfate and generate the necessary energy for its survival.^43,44^ Note that when hydrogen is used as the reductant, a separate carbon source would be required to sustain the microbial growth, which has not been considered in this modeling study.

For both cycles, our simulations showed ∼5.5 g of CO_2_/g of H_2_ being captured from air through MgCO_3_ precipitation. With the fast-developing renewable energy technologies, the International Energy Agency (IEA) projected a carbon footprint of H_2_ supply as low as 1 kg CO_2_-eq/kg H_2_ by 2050.^45^ This would allow the simulated system to achieve net CDR. However, there is still significant scope for exploring alternative metabolic pathways to allow the desirable pH swing to be implemented with electron donors of a much lower energy cost, which is imperative to improve the competitiveness of this scheme as a CDR option. Furthermore, the energy consumption by the other aspects of operating the sulfur-cycle system (including stirring and mineral grinding), which has not been considered in this work, needs to be assessed and minimized. Finally, although considerations were given in this work to the implications of the choice between different electron donors, a more comprehensive study will need to fully quantify the net CO_2_ removal potential of each choice, with a scope covering not only the operation of the bioreactors but also the rest of the whole life cycle, including particularly the supply of materials and energy.

#### Choice of Alkaline Mineral

In this study, forsterite was selected as the alkaline mineral for dissolution, which was guided by following critical considerations: (1) the mineral should have a relatively fast dissolution rate in an acidic environment; (2) the dissolution process should release metal ions that do not form a significant amount of precipitates with sulfate ions as this would interrupt the intended sulfur cycle; (3) released metal ions can react with CO_2_ in the reduction bioreactor to form insoluble precipitates in water; and (4) the mineral is globally abundant (e.g., produced in industrial scale operations such as mining, with additional possibilities to incorporate systems into existing infrastructure for large-scale removal opportunities).^17,18^

While forsterite meets the above criteria well, considerations were also given to other silicates in this work, including particularly calcium-containing silicate minerals, such as wollastonite, which has been previously targeted for CDR in carbonation studies.^46,47^ In connection with a pH swing-based system, wollastonite demonstrates a high dissolution rate in an acidic environment (eq 27). Its dissolution releases calcium ions, which can react with atmospheric CO_2_ to form stable calcium carbonate precipitates (eq 28), which thus represents a potentially effective process of capturing and storing CO_2_ from the air^46,47^
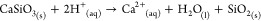
27
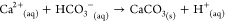
28A key limitation to this approach is that wollastonite and other calcium-containing silicate minerals are not well suited for sulfur-cycle-based carbon mineralization studied in this work. This is because, within the oxidation bioreactor, calcium ions would react with sulfate ions in the solution to form calcium sulfate (CaSO_4_). CaSO_4_ is poorly soluble in water, with a solubility of only about 0.16 mol per liter of water at 25 °C, which is less than 1% of the solubility of magnesium sulfate (MgSO_4_) under the same conditions.^48,49^ Within the range of concentrations considered in this study, the use of wollastonite for dissolution would lead to the precipitation of CaSO_4_ in the oxidation bioreactor. This would result in the loss of sulfur from the sulfur cycle and loss of calcium ions for forming CaCO_3_, thus diminishing the system’s potential for capturing CO_2_ from the atmosphere.

## Conclusions

This study has explored a sulfur-cycle-based approach to implement pH swing that enables the accelerated dissolution of silicate minerals and the subsequent carbonation to remove atmospheric CO_2_. The assessment of the process concept was carried out through mathematically modeling two alternative cycles, which differ in the reduced form of sulfur. The simulation results have shown that both the hydrogen sulfide (H_2_S) cycle and the thiosulfate (S_2_O_3_^2–^) cycle can successfully achieve pH swings under ambient temperature and pressure and facilitate the release of magnesium ions from forsterite and the removal of CO_2_ from air in the form of magnesium carbonate (MgCO_3_) as a solid product. The use of thiosulfate, which avoids the transfer of a highly corrosive and toxic gas between the two bioreactors, represents a better option than hydrogen sulfide for implementing a closed sulfur cycle. The major rate-limiting processes for both options are forsterite dissolution in the oxidation bioreactor and the gas–liquid mass transfer of CO_2_ in the reduction bioreactor, which should be the targets for future improvements along with the reduction of energy cost for supplying a low-carbon electron donor to the reduction reactor. Such improvements could pave the way for future scaling-up of bioreactor-based CDR methods, allowing for integration into established industrial frameworks, such as mining or steelmaking operations. This integration holds promise for meaningful contributions toward global climate targets through efficient large-scale implementations of permanent CO_2_ removal.

## Supporting Information Available

Model parameters; calculation of mass-transfer coefficient (*k*_L_*a*), fractional gas hold-up (ε_G_), and enhancement factor (*E*); composition of the culture medium and mine tailing; sensitivity analysis; gas and liquid-phase mass balance; and aqueous phase CO_2_–H_2_O equilibrium and charge balance (PDF)
