# Supplementary material for: Modeling and Feasibility Assessment of Mineral Carbonation Based on Biological pH Swing for Atmospheric CO2 Removal
Source: ACS Sustain Chem Eng. 2025 May 8;13(19):6972–81. doi: 10.1021/acssuschemeng.4c10708 (PMC12093374; doi:10.1021/acssuschemeng.4c10708)
Supplement: Supplementary file 1 — sc4c10708_si_001.pdf [file sc4c10708_si_001.pdf]

Supplementary Information

Modelling and Feasibility Assessment of Mineral Carbonation Based on Biological pH Swing for Atmospheric CO_2 Removal

Yukun Zhang [a], Spencer Long [b], Manon T Duret [c, d], Liam A. Bullock [e], Phyllis Lam [b,*], Aidong Yang [a,*]

[a] Department of Engineering Science, University of Oxford, Oxford, OX1 3PJ, UK

[b] School of Ocean and Earth Science, University of Southampton, Southampton, SO14 3ZH, UK

[c] Department of the Geophysical Sciences, University of Chicago, IL, USA

[d] Climate Systems Engineering Initiative, University of Chicago, IL, USA

[e] Geological and Mining Institute of Spain, IGME C/Rios Rosas 23, Madrid, 28003, Spain

[*] Corresponding authors. E-mails: p.lam@southampton.ac.uk; aidong.yang@eng.ox.ac.uk

Number of pages: 11
Number of figures: 4
Number of tables: 7

Supplementary Data

S1. Model parameters

Table S1. Model parameters for oxidation bioreactor.

NOTATION	PARAMETER	VALUE	UNIT	SOURCE
μ_{max}	Maximum specific growth rate of *A. thiooxidans*	0.037	h^{-1}	[1]
k_d	Decay rate of *A. thiooxidans*	0.003	h^{-1}	Assumed
K_S	Half saturation constant	0.150	mg L^{-1}	[1]
K_O	Kinetic constant of oxygen for microbial growth	1.100	mg L^{-1}	[1]
H_S	Henry's law constant for H_2S	0.427	-	[2]
H_O	Henry's law constant for oxygen	32.300	-	[2]
N	Impeller speed	1.667	rev s^{-1}	Assumed
D	Impeller diameter	0.376	m	Assumed
D_I	Width of the impeller blade	0.075	m	Assumed
V_L	Liquid volume of reactor	1.000	m^3	Assumed
μ_L	Liquid viscosity	0.001	Pa s	-
Y_{XS}	Yield coefficient for oxidation	0.093	-	[1]
M	Molar mass of forsterite	1.41E+05	mg mol^{-1}	[3]
ρ_f	Average density of forsterite particles	3.27E+09	mg m^{-3}	[3]

Table S2. Model parameters for reduction bioreactor.

NOTATION	PARAMETER	VALUE	UNIT	SOURCE
H_H	Henry constant for hydrogen	1.364	-	[4]
$Y_{H_2S,S}$	Moles of sulphate ion would be needed to generate one mole of H_2S ions	1	-	-
$Y_{S_2O_3,S}$	Moles of sulphate ion would be needed to generate one mole of $S_2O_3^{2-}$ ions	0.5	-	-
H_C	Henry's law constant for CO_2	28.6651	atm L mol^{-1}	[5]
D_L	Molecular diffusivities of CO_2 in liquid	1.92E-09	m^2 s^{-1}	[6]
σ	Surface tension of liquid	0.072	N m^{-1}	[7]
$K_{H_2S,aq}$	Equilibrium constant	10E+6.99	-	[8]
$K_{H_2S,g}$	Equilibrium constant	1.00E-07	-	[8]
M_{MgCO_3}	Molar mass for $MgCO_3$	84.3139	g mol^{-1}	[9]
ρ_{MgCO_3}	Density for $MgCO_3$	2.96E+06	g m^{-3}	[9]

S2. Composition of the culture medium

To support the growth of *A. thiooxidans*, we prepared a medium and adjusted its pH to a range between 4.4 and 4.7. Subsequently, the medium was autoclaved at 121 Celsius degrees for 15 minutes. The composition of the medium is as follows:

Table S3. Composition of the medium prepared for A. thiooxidans.

Chemicals	Amount	Unit
KH_2PO_4	3	g
$MgSO_4$ x 7 H_2O	0.5	g
$(NH_4)_2SO_4$	3	g
$CaCl_2$ x 2 H_2O	0.25	g
$Na_2S_2O_3$ x 5 H_2O	5	g
Distilled water	1000	ml

S3. Minerals in the tailing

Table S4. Dissolution reactions and parameters for different mineral in the mine tailings [14].

Mineral Name	Dissolution reaction	A (mol m^{-2} h^{-1})	E (J mol^{-1})	n (-)
Plagioclase	Plagioclase + $8H^+ \leftrightarrow 2Al^{3+} + Ca^{2+} + 2H_4SiO_4$	9.29E+02	1.66E+04	1.41
Clinopyroxene	Clinopyroxene + $4H^+ \leftrightarrow Mg^{2+} + Ca^{2+} + 2H_4SiO_4$	7.31E+07	8.80E+04	0.50
Orthopyroxene	Orthopyroxene + $2H^+ + H_2O \leftrightarrow Mg^{2+} + H_4SiO_4$	3.60E+08	8.00E+04	0.60
Serpentine	Serpentine + $6H^+ \leftrightarrow 3Mg^{2+} + 2H_4SiO_4 + H_2O$	7.20E+05	7.35E+04	0.21
Mica	Mica + $8H^+ \leftrightarrow Al^{3+} + K^+ + 3Mg^{2+} + 3H_4SiO_4$	4.51E+13	8.50E+04	0.7
Calcite	Calcite + $H^+ \leftrightarrow Ca^{2+} + HCO_3^-$	3.13E+28	1.44E+05	1
Dolomite	Dolomite + $2H^+ \leftrightarrow Ca^{2+} + Mg^{2+} + 2HCO_3^-$	4.93E+6	3.61E+04	0.5

Table S5. Abundance of minerals in the mine tailings.

Mineral	Formulae	Modal abundance (%)
**Amphibole**	$Ca_2(Mg,Fe,Al)_5(Al,Si)_8O_{22}(OH)_2$	6
**Calcite**	$CaCO_3$	1
**Plagioclase**	$CaAl_2Si_2O_8$	17
**Clinopyroxene**	$MgCaSi_2O_6$	17
**Orthopyroxene**	$MgSiO_3$	25
**Quartz**	SiO_2	3
**Chlorite**	$Mg_5Al(AlSi_3O_{10})(OH)_8$	6
**Serpentine**	$Mg_3(Si_2O_5)(OH)_4$	12

**Mica**	$Ca(Mg,Al)_3(Al_3Si)O_{10}(OH)_2$	4
**Talc**	$Mg_3Si_4O_{10}(OH)_2$	8
**Dolomite**	$CaMg(CO_3)_2$	1

S4. The initial conditions for simulations

Table S6. The initial conditions adopted for each simulation run in the oxidation bioreactor.

	Oxidation Bioreactor			
	Forsterite (mg L^{-1})	C_{LS} (mg L^{-1})	C_{LO} (mg L^{-1})	Microbe density (mg L^{-1})
**H_2S cycle**	200000	0	5.9	1000
**S_2O_3 cycle**	200000	124050	5.9	1000

Table S7. The initial conditions adopted for each simulation run in the reduction bioreactor.

	Reduction Bioreactor		
	Mg^{2+} ions (mol L^{-1})	SO_4^{2-} ions (mol L^{-1})	Microbe density (mg L^{-1})
**H_2S cycle**	0.5	0.5	20
**S_2O_3 cycle**	1	1	20

S5. Sensitivity analysis

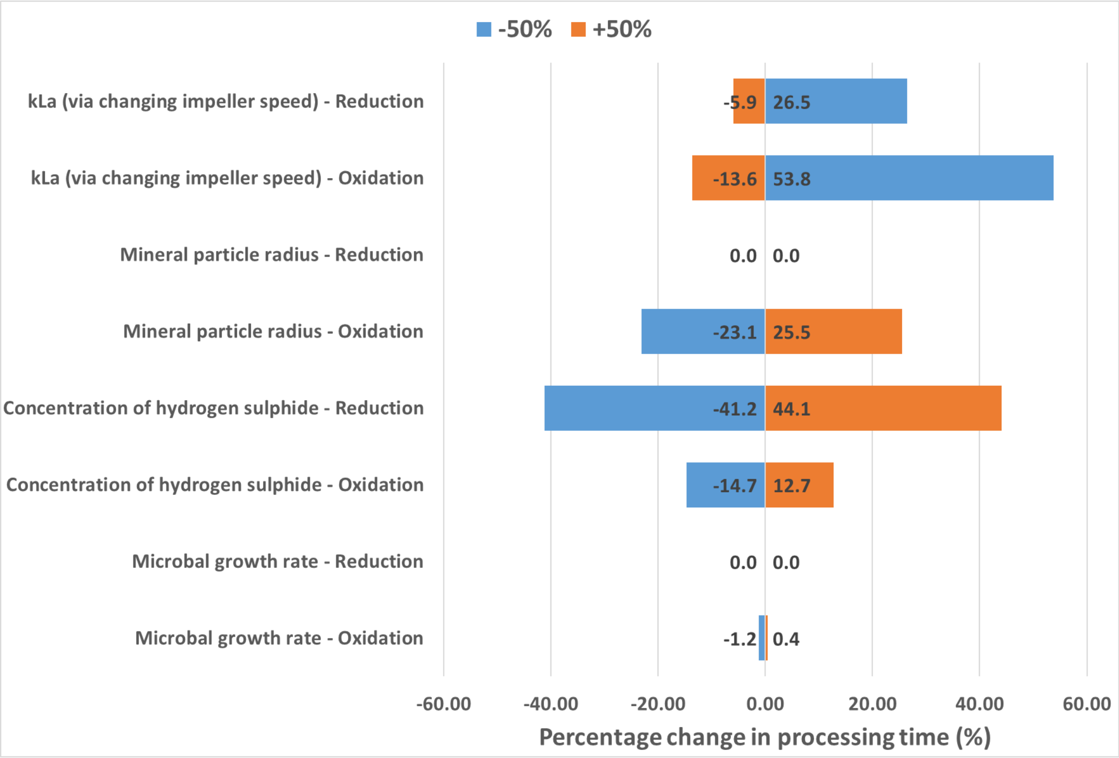

Fig. S1. Sensitivity analysis for mineral carbonation efficiency in hydrogen sulphide cycle.

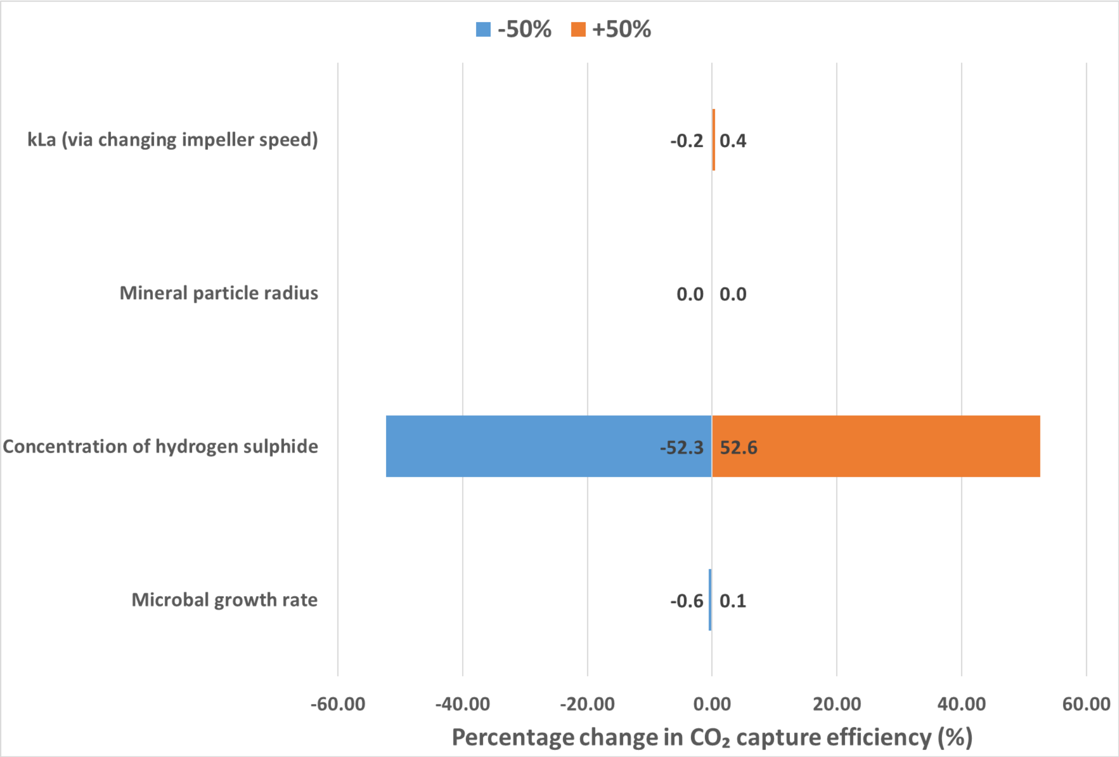

Fig. S2. Sensitivity analysis for CO_2 capture efficiency in hydrogen sulphide cycle.

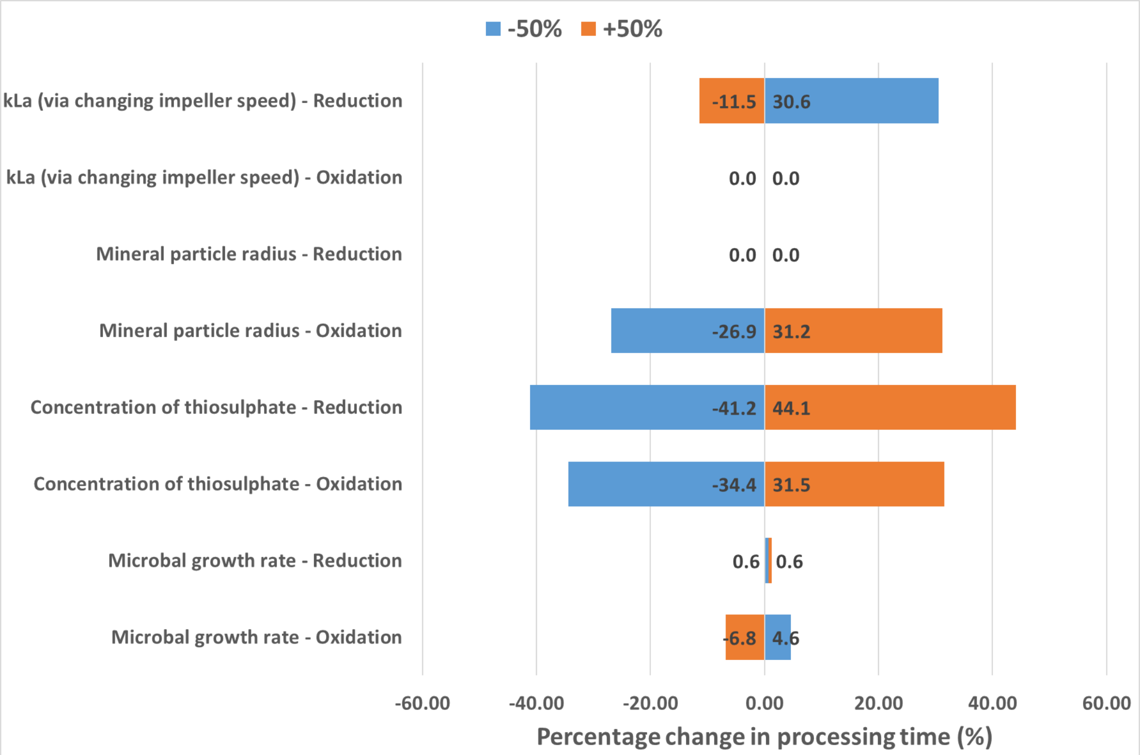

Fig. S3. Sensitivity analysis for mineral carbonation efficiency in thiosulphate cycle.

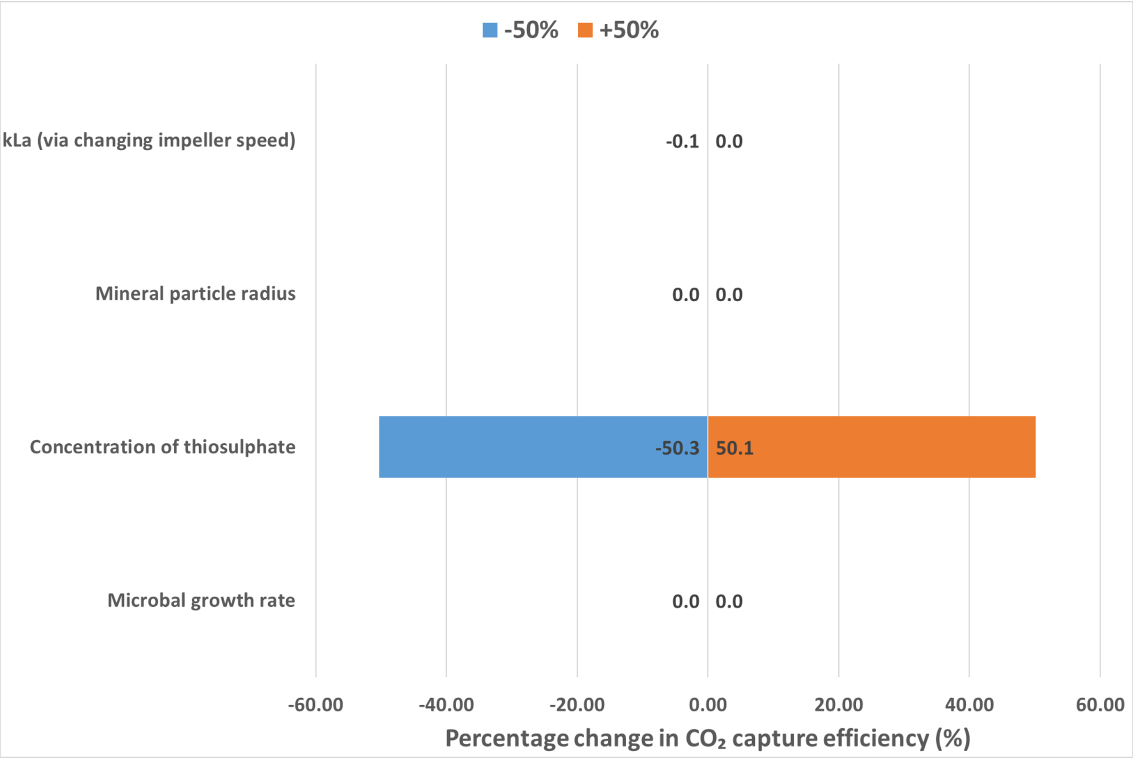

Fig. S4. Sensitivity analysis for CO_2 capture efficiency in thiosulphate cycle.

S6. Calculation of $k_L a$

$$k_L a = 6.48 * \left(\frac{N}{N_{cd}}\right)^{1.44} * v_G{}^{1.12} \quad \text{(S1)}$$

$$N_{cd} = \frac{4 * \mathrm{Q}_G{}^{0.5} * D^{0.25}}{D_I{}^2} \quad \text{(S2)}$$

As shown in Eqs. (S1) and (S2), we apply the method introduced by Smith to estimate the $k_L a$ value in stirred tank reactors [10, 11]. In these equations, N (rev s^{-1}) refers to impeller speed, N_{cd} (rev s^{-1}) refers to the minimum impeller speed for complete suspension of all the solid particles in stirred tank reactors, v_G (m s^{-1}) is gas velocity, Q_G (L h^{-1}) is volumetric gas flow rate, D and D_I (m) refer to diameters of the reactor and the impeller, respectively.

Note that for simulating the experimental oxidation reactor, as there was no active gas supply to the reactor, the gas phase mass balance for O_2 was excluded from the bioreactor model, while a modest $k_L a$ value (0.4 h^{-1}) was assumed to approximate the mass transfer of oxygen into the culture medium at the top of the reactor.

S7. Calculation of ε_G

$$\varepsilon_G = \frac{V_G}{V_R} = \left(\frac{1.98 * v_G}{1 + 40.9 * v_G}\right) * \left(\frac{P_G}{V_L}\right)^{0.21} \quad \text{(S3)}$$

$$P_G = 0.1 * P_0 * \left(\frac{Q_G}{N*V_L}\right) * \left(\frac{N^2*D^4}{g*D_i*V_L^{\frac{2}{3}}}\right)^{-0.2} \quad \text{(S4)}$$

In Eq. (S3) and (S4), ε_G (-) refers to fractional gas hold up, P_G (W) refers to power consumption and P_0 (W) refers to power consumption in absence of gas. We can calculate P_0 by using following equations [12, 13]:

$$P_0 = \rho_L * N^3 * D^5 * N_p \quad \text{(S5)}$$

$$N_p = \min\left(19.5 * Re_L{}^{-0.3}; 24 * (Re_L * Fr_L)^{-\frac{1}{3}}\right) \quad \text{(S6)}$$

$$Re_L = \frac{\rho_L*N*D^2}{\mu_L} \quad \text{(S7)}$$

$$Fr_L = \frac{D*N^2}{g} \quad \text{(S8)}$$

In Eq. (S5), (S6), (S7) and (S8), ρ_L (kg m^{-3}) is density of liquid, N_p (-) is power number, Re_L (-) is Reynolds number, Fr_L (-) is Froude number, μ_L (Pa s) is liquid viscosity and g (m s^{-2}) is acceleration of gravity.

S8. Calculation of *E*

$$E = 1 + \left[\left(\frac{H_C*1.67C_{\mathrm{OH^-}}}{2C_{GC,in}}\right)^{-1.35} + \left(\frac{\sqrt{D_L k_2 C_{\mathrm{OH^-}}}}{k_L \tanh\left(\frac{\sqrt{D_L k_2 C_{\mathrm{OH^-}}}}{k_L}\right)} - 1\right)^{-1.35}\right]^{-1.35} \quad \text{(S9)}$$

$$\log k_2 = 11.895 - \frac{2382}{T} + 0.221 I_c - 0.016 I_c^2 \quad \text{(S10)}$$

$$k_L = \frac{k_L a_{CO_2}}{1.38*\left(\frac{g*\rho_L}{\sigma}\right)^{0.5} * \left(\frac{Q_G}{N*V_L}\right)^{\frac{1}{3}} * \left(\frac{N^2*D^4}{g*D_i*V_L^{\frac{2}{3}}}\right)^{0.592} * \left(\frac{D_b*N^2*D^4}{\sigma*V_L^{\frac{2}{3}}}\right)^{0.187}} \quad \text{(S11)}$$

$$D_b = \frac{5.5*\sigma*\varepsilon_G^{\frac{1}{2}}}{g*\rho_L*\left(\frac{N^2*D^3}{g*D_i*V_L^{\frac{2}{3}}*\left(\frac{P_G}{P_0}\right)^{\frac{2}{3}}}\right)^{\frac{1}{2}}} \quad \text{(S12)}$$

We apply Eq. (S9 – S12) to calculate enhancement factor E. In these equations, $C_{\mathrm{OH^-}}$ (mol L^{-1}) refers to concentration of hydroxide in liquid, $C_{GC,in}$ (atm) refers to concentration of CO_2 in supplied air, D_L (m^2 s^{-1}) refers to molecular diffusivities of CO_2 in liquid, I_c (mol L^{-1}) refers to ionic strength, σ (N s^{-1}) refers to surface tension of liquid, D_b (m) refers to diameter of bubbles.

S9. Liquid phase mass balance in the oxidation bioreactor

$$r_{G-L,S} = k_L a_S * \left(\frac{C_{GS}}{H_S} - C_{LS}\right) \quad \text{(S13)}$$

$$r_{G-L,O} = k_L a_O * \left(\frac{C_{GO}}{H_O} - C_{LO}\right) \quad \text{(S14)}$$

In Eqs. (S13) (only applicable to H_2S, not to $S_2O_3^{2-}$) and (S14), $r_{G-L,S}$ and $r_{G-L,O}$ (mg L^{-1}) refer to the rates of dissolution for H_2S and oxygen; k_La_S and k_La_o (h^{-1}) represent volumetric mass-transfer coefficient for H_2S and oxygen; C_{GS} and C_{GO} (mg L^{-1}) refer to the concentrations of H_2S and oxygen in gas; H_S and H_O (-) refer to Henry's law constant for H_2S and oxygen. The k_La values are calculated using previously established correlations for aerated and agitated reactors; details are provided in the section S6.

Eqs. (S15) and (S16), supported by Eq. (S17), represent the rates of change in the concentrations of dissolved H_2S and DO, respectively:

$$\frac{dC_{LS,H_2S}}{dt} = r_{G-L,S} * \frac{V_R}{V_L} - r_{S,H_2S} \quad \text{(S15)}$$

$$\frac{dC_{LO}}{dt} = r_{G-L,O} * \frac{V_R}{V_L} - r_O \quad \text{(S16)}$$

$$V_R = V_L + V_G \quad \text{(S17)}$$

V_R, V_L and V_G (L) represent the total reaction volume of the bioreactor (excluding the volume of the solid particles), liquid volume and gas volume, respectively. r_{S,H_2S} (mg L^{-1}) refers to rate of H_2S oxidation by *A. thiooxidans*, and r_O (mg L^{-1}) refers to microbial consumption of oxygen in liquid, which could be calculated by the following Eqs. (S18) and (S19):

$$r_S = \frac{\mu_{max} * X * \gamma}{Y_{XS}} \quad \text{(S18)}$$

$$r_O = \frac{\mu_{max} * X * \gamma}{Y_{XO}} \quad \text{(S19)}$$

In these two equations, Y_{XS} (-) refers to the yield coefficient for oxidation of H_2S or $S_2O_3^{2-}$. Unlike H_2S, the supply of $S_2O_3^{2-}$ ions do not involve gas-liquid mass transfer from gas to liquid; therefore, the change in concentration of $S_2O_3^{2-}$ ions can be represented with following modified equation, as opposed to Eq. (S15):

$$\frac{dC_{LS,S2O3}}{dt} = - r_{s,S2O3} \quad \text{(S20)}$$

In this equation, $C_{LS,S2O3}$ (mg L^{-1}) refers to concentration of $S_2O_3^{2-}$ ions, and $r_{s,S2O3}$ (mg L^{-1}) refers to rate of $S_2O_3^{2-}$ oxidation by *A. thiooxidans*, which can be calculated by applying Eq. (S18) to thiosulphate.

S10. Gas phase mass balance in the oxidation bioreactor

For mass balance in the gas phase, Eq. (S21) and (S22) correspondingly represent the rates of change in concentrations of gaseous H_2S and oxygen:

$$\frac{dC_{GS,H_2S}}{dt} = \frac{Q_G * (C_{GS,in} - C_{GS,H_2S})}{V_G} - \frac{r_{G-L,S}}{\varepsilon_G} \quad \text{(S21)}$$

$$\frac{dC_{GO}}{dt} = \frac{Q_G * (C_{GO,in} - C_{GO})}{V_G} - \frac{r_{G-L,S}}{\varepsilon_G} \quad \text{(S22)}$$

In these equations, Q_G (L h^{-1}) refers to gas flow rate in the bioreactor, $C_{GS,in}$ (mg L^{-1}) refers to concentration of H_2S in feed gas, V_G (L) refers to effective gas volume, and ε_G (-) refers to fractional gas hold up; details of ε_G calculation are provided in section S7.

S11. Liquid phase mass balance in the reduction bioreactor

For simplicity, the hydrogen supply in the reactor was assumed to be via a liquid phase that was in equilibrium with a hydrogen gas phase at 1 bar, using Eq. (S23) to simulate the concentration of aqueous hydrogen (C_{LH}) in the reduction bioreactor:

$$C_{LH} = C_{GH} * H_H \quad \text{(S23)}$$

where C_{GH} (atm) refers to the concentration of H_2 in gas, H_H (mol L^{-1} atm^{-1}) refers to the Henry constant for H_2. Note that the supply of hydrogen takes place at a stage different from (more precisely, earlier than) that for the supply of air and removal of H_2S (in the sulphide cycle), thus the operation of the reactor does not involve mixing H_2 with any gas flow through the reactor and avoids the loss of H_2.

For concentrations of reduced sulphur compounds (C_{LS}), due to the different natures of hydrogen sulphide and thiosulphate, we apply separate equations to simulate the rates of change in concentrations:

$$\frac{dC_{LS,H_2S}}{dt} = -Y_{H_2S,S} * r_S - r_{L-G,H_2S} * \frac{V_R}{V_L} \quad \text{(S24)}$$

$$\frac{dC_{LS,S2O3}}{dt} = -Y_{S2O3,S} * r_S \quad \text{(S25)}$$

$$r_{L-G,H_2S} = C_{GS,H_2S} * Q \quad \text{(S26)}$$

where $Y_{H_2S,S}$ and $Y_{S_2O_3,S}$ (-) correspondingly refer to how many moles of H_2S or $S_2O_3^{2-}$ ions would be generated when one mole of sulphate ions are reduced, r_{L-G,H_2S} (mol h^{-1}) refers to the rate of H_2S gas released from liquid, C_{GS,H_2S} (mol L^{-1}) refers to concentration of H_2S in the gas phase, and Q (L h^{-1}) refers to air flow rate in the reduction bioreactor. For simplicity, chemical and phase equilibria are assumed for the H_2S-H_2O system, which allows C_{GS,H_2S} to be determined based on the concentration of total dissolved H_2S in the liquid phase, C_{LS,H_2S} (further explained in Section S12).

We use Eq. (S27) to simulate the change in liquid phase CO_2 concentration:

$$\frac{dC_{LC}}{dt} = r_{G-L,CO_2} * \frac{V_R}{V_L} - r_{cap} \quad \text{(S27)}$$

where r_{cap} (mol L^{-1} h^{-1}) refers to the rate of CO_2 capture. For the replenishment rate of CO_2 in the liquid phase, r_{G-L,CO_2} (mol L^{-1} h^{-1}), we apply Eq. (S28).

$$r_{G-L,CO_2} = k_L a_{CO_2} * E * \left(\frac{C_{GC}}{H_C} - C_{LC}\right) \quad \text{(S28)}$$

In this equation, $k_L a_{CO_2}$ (h^{-1}) refers to the volumetric mass-transfer coefficient value for CO_2, C_{GC} (atm) and C_{LC} (mol L^{-1}) correspondingly refer to the concentration of CO_2 in gas and liquid, H_C (atm L mol^{-1}) refers to Henry's law constant for CO_2, E (-) is the enhancement factor to account for the chemical effect on mass transfer [12, 15]. The details for calculating E are provided section S8.

S12. Gas phase mass balance in the reduction bioreactor

H_2S can be present the reactor in both gaseous and dissolved states, with their ratio depending on the pH of the liquid phase. The split of H_2S in the two phases is modelled based on phase and dissociation equilibria:

$$C_{LS,H_2S} = C_{H_2S,aq} + C_{HS^-} \quad \text{(S29)}$$

$$C_{H_2S,aq} = K_{H_2S,aq} * C_{HS^-} * 10^{-pH} \quad \text{(S30)}$$

$$C_{GS,H_2S} = C_{HS^-} * \frac{10^{-pH}}{K_{H_2S,g}} \quad \text{(S31)}$$

where C_{GS,H_2S} is the concentration of gaseous H_2S, C_{HS^-} and $C_{H_2S,aq}$ (mol L^{-1}) refer to concentrations of HS^- ions and H_2S in solution, $K_{H_2S,aq}$ and $K_{H_2S,g}$ (-) refer to equilibrium constants for two reactions. The gaseous portion of H_2S will be removed with the air flowing through the reactor; this gas mixture subsequently feeds into the oxidation bioreactor.

For CO_2, we apply Eq. (S32) to simulate its gas phase concentration in reduction bioreactor:

$$\frac{dC_{GC}}{dt} = \frac{Q_G*(C_{GC,in} - C_{GC})}{V_G} - \frac{r_{G-L,CO_2}}{\varepsilon_G} \quad \text{(S32)}$$

S13. Aqueous phase CO_2-H_2O equilibrium and charge balance

Complementary to the model equations introduced in sections 2.2 and 2.3, the model for each bioreactor further includes the following chemical reaction equilibria:

$$CO_2 + H_2O \leftrightarrow HCO_3^- + H^+ \quad \text{(S33)}$$

$$HCO_3^- \leftrightarrow CO_3^{2-} + H^+ \quad \text{(S34)}$$

$$H_2O \leftrightarrow H^+ + OH^- \quad \text{(S35)}$$

Finally, charge balance is included to complete the model, particularly to allow pH to be calculated:

$$\sum_i (C_{cation_i} * z_i) + \sum_j (C_{anion_j} * z_j) = 0 \quad \text{(S36)}$$

In this equation, C_{cation_i} and C_{anion_j} (mol L^{-1}) refer to concentration of specific cation or anion respectively; z_i and z_j (C) refer to the charge number for the cation or anion.

References

1. Namgung, H.-K. and J. Song *The Effect of Oxygen Supply on the Dual Growth Kinetics of*

Acidithiobacillus thiooxidans under Acidic Conditions for Biogas Desulfurization. International Journal of Environmental Research and Public Health, 2015. **12**, 1368-1386 DOI: 10.3390/ijerph120201368.

2. Benjamin, M.M. and D.F. Lawler, *Water quality engineering : physical/chemical treatment processes*. 2013, Wiley: Hoboken, New Jersey.

3. *Webmineral: Forsterite Mineral Data*. Available from: https://webmineral.com/data/Forsterite.shtml.

4. Sander, R., *Compilation of Henry's law constants (version 4.0) for water as solvent.* Atmospheric Chemistry and Physics, 2015. **15**(8): p. 4399-4981.

5. Sander, R., *Compilation of Henry's law constants (version 5.0.0) for water as solvent.* Atmos. Chem. Phys., 2023. **23**(19): p. 10901-12440.

6. Cussler, E.L., *Diffusion: mass transfer in fluid systems*. 2009: Cambridge university press.

7. Hauner, I.M., et al., *The Dynamic Surface Tension of Water.* The Journal of Physical Chemistry Letters, 2017. **8**(7): p. 1599-1603.

8. *Thermoddem*. Available from: https://thermoddem.brgm.fr/.

9. Centers for Disease, C., *NIOSH pocket guide to chemical hazards*. 1990: National Institute for Occupational Safety and Health.

10. Smith, J.M., *Simple Performance Correlations for Agitated Vessels*, in *Fluid Mechanics of Mixing: Modelling, Operations and Experimental Techniques*, R. King, Editor. 1992, Springer Netherlands: Dordrecht. p. 55-63.

11. Yawalkar, A.A., et al., *Gas—Liquid Mass Transfer Coefficient in Stirred Tank Reactors.* The Canadian Journal of Chemical Engineering, 2002. **80**(5): p. 840-848.

12. Hughmark, G.A., *Power requirements and interfacial area in gas-liquid turbine agitated systems.* Industrial & Engineering Chemistry Process Design and Development, 1980. **19**(4): p. 638-641.

13. Scargiali, F., et al., *Power Consumption in Uncovered Unbaffled Stirred Tanks: Influence of the Viscosity and Flow Regime.* Industrial & Engineering Chemistry Research, 2013. **52**: p. 14998-15005.

14. James L. Palandri, Y.K.K., *A COMPILATION OF RATE PARAMETERS OF WATER-MINERAL INTERACTION KINETICS FOR APPLICATION TO GEOCHEMICAL MODELING*. 2004, National Energy Technology Laboratory – United States Department of Energy: Menlo Park, California.

15. Wellek, R.M., R.J. Brunson, and F.H. Law, *Enhancement factors for gas-absorption with second-order irreversible chemical reaction.* The Canadian Journal of Chemical Engineering, 1978. **56**(2): p. 181-186.
